# Substantia Nigra MRI markers are lower in Alzheimer's disease and are linked to general cognitive function

**DOI:** 10.1093/braincomms/fcag238

**Published:** 2026-06-24

**Authors:** Friedrich Krohn, Mousumi Sarkar, Hartmut Schütze, Panagiotis Iliopoulos, Lucía Penalba-Sánchez, Dorothea Hämmerer, Renat Yakupov, Falk Lüsebrink, Annika Spottke, Anja Schneider, Nina Roy, Enise Incesoy, Michael Heneka, Ingo Kilimann, Luca Kleineidam, Stefan J Teipel, Frederic Brosseron, Doreen Goerss, Wenzel Glanz, Matthias Schmid, Ayda Rostamzadeh, Michael Wagner, Klaus Fliessbach, Frank Olaf Jessen, Emrah Düzel, Matthew J Betts

**Affiliations:** Institute of Cognitive Neurology and Dementia Research (IKND), Otto-von-Guericke University, Magdeburg 39120, Germany; Institute of Cognitive Neurology and Dementia Research (IKND), Otto-von-Guericke University, Magdeburg 39120, Germany; Institute of Cognitive Neurology and Dementia Research (IKND), Otto-von-Guericke University, Magdeburg 39120, Germany; Institute of Cognitive Neurology and Dementia Research (IKND), Otto-von-Guericke University, Magdeburg 39120, Germany; Institute of Cognitive Neurology and Dementia Research (IKND), Otto-von-Guericke University, Magdeburg 39120, Germany; Betts Group, German Center for Neurodegenerative Diseases, Magdeburg 39120, Germany; Institute of Cognitive Neurology and Dementia Research (IKND), Otto-von-Guericke University, Magdeburg 39120, Germany; Betts Group, German Center for Neurodegenerative Diseases, Magdeburg 39120, Germany; Institute of Cognitive Neurology and Dementia Research (IKND), Otto-von-Guericke University, Magdeburg 39120, Germany; Betts Group, German Center for Neurodegenerative Diseases, Magdeburg 39120, Germany; Institute of Cognitive Neurology and Dementia Research (IKND), Otto-von-Guericke University, Magdeburg 39120, Germany; Leitung klinische Forschung, German Center for Neurodegenerative Diseases, Bonn 53127, Germany; Leitung klinische Forschung, German Center for Neurodegenerative Diseases, Bonn 53127, Germany; Department of Old Age Psychiatry and Cognitive Disorders, 53127 University Hospital Bonn, Bonn, Germany; Leitung klinische Forschung, German Center for Neurodegenerative Diseases, Bonn 53127, Germany; Department of Old Age Psychiatry and Cognitive Disorders, 53127 University Hospital Bonn, Bonn, Germany; Institute of Cognitive Neurology and Dementia Research (IKND), Otto-von-Guericke University, Magdeburg 39120, Germany; Betts Group, German Center for Neurodegenerative Diseases, Magdeburg 39120, Germany; Department for Psychiatry and Psychotherapy, University Clinic Magdeburg, 39120 Magdeburg, Germany; Leitung klinische Forschung, German Center for Neurodegenerative Diseases, Bonn 53127, Germany; Luxembourg Centre for Systems Biomedicine (LCSB), University of Luxembourg, Belvaux 4367, Luxembourg; Klinische Demenzforschung, German Center for Neurodegenerative Diseases, Rostock 18147, Germany; Leitung klinische Forschung, German Center for Neurodegenerative Diseases, Bonn 53127, Germany; Department of Old Age Psychiatry and Cognitive Disorders, 53127 University Hospital Bonn, Bonn, Germany; Klinische Demenzforschung, German Center for Neurodegenerative Diseases, Rostock 18147, Germany; Leitung klinische Forschung, German Center for Neurodegenerative Diseases, Bonn 53127, Germany; Leitung klinische Forschung, German Center for Neurodegenerative Diseases, Bonn 53127, Germany; Department of Old Age Psychiatry and Cognitive Disorders, 53127 University Hospital Bonn, Bonn, Germany; Institute of Cognitive Neurology and Dementia Research (IKND), Otto-von-Guericke University, Magdeburg 39120, Germany; Betts Group, German Center for Neurodegenerative Diseases, Magdeburg 39120, Germany; Institute for Medical Biometry, Informatics and Epidemiology, Medical Faculty, University of Bonn, Bonn 53127, Germany; Department of Psychiatry, Medical Faculty, University of Cologne, Cologne 50924, Germany; Leitung klinische Forschung, German Center for Neurodegenerative Diseases, Bonn 53127, Germany; Department of Old Age Psychiatry and Cognitive Disorders, 53127 University Hospital Bonn, Bonn, Germany; Leitung klinische Forschung, German Center for Neurodegenerative Diseases, Bonn 53127, Germany; Department of Old Age Psychiatry and Cognitive Disorders, 53127 University Hospital Bonn, Bonn, Germany; Faculty of Medicine, University of Cologne, Cologne 50932, Germany; Institute of Cognitive Neurology and Dementia Research (IKND), Otto-von-Guericke University, Magdeburg 39120, Germany; Betts Group, German Center for Neurodegenerative Diseases, Magdeburg 39120, Germany; Institute of Cognitive Neurology and Dementia Research (IKND), Otto-von-Guericke University, Magdeburg 39120, Germany; Betts Group, German Center for Neurodegenerative Diseases, Magdeburg 39120, Germany

**Keywords:** Substantia Nigra (SN), Alzheimer's disease, neuromelanin, memory, novelty

## Abstract

Individuals with Alzheimer’s disease dementia show Alzheimer's disease pathology and a heterogeneous degeneration of the Substantia Nigra (SN) post-mortem. However, it is unclear how SN degeneration is related to cognitive dysfunction across the Alzheimer’s disease dementia continuum. In this study, using data from the prospective DZNE-Longitudinal Cognitive Impairment and Dementia Study (DELCODE), we investigated whether *in vivo* SN MRI measures are lower in individuals with clinically defined Alzheimer’s disease dementia than in healthy control subjects (HC) and if they are associated with hippocampal functional activity during the processing of novel visual stimuli and subsequent recognition memory. One hundred and sixty DELCODE participants (69 years ± 6 years, 88 men), including 79 HC, 70 individuals with subjective cognitive decline (SCD), 17 individuals with mild cognitive impairment (MCI) and 10 individuals with Alzheimer’s disease dementia, completed a scene novelty and encoding task and a 3T SN-sensitive MRI scan, from which the two *in vivo* SN measures MRI contrast and volume were calculated and harmonized between scanner sites while preserving diagnostic group differences. For 71 individuals, CSF levels of phosphoTau, total tau and amyloid-beta 42/40 ratio (Aß42/40) were available. All individuals completed a neuropsychological task battery from which a global cognitive score was calculated. In separate models, we assessed the relationship between SN MRI markers and CSF levels of Alzheimer’s disease, the global cognitive score, hippocampal novelty activation and recognition memory while accounting for age, sex, years of education and total intracranial volume (TIV). SN volume but not SN MRI contrast was lower in individuals with clinical Alzheimer’s disease dementia [one-way analyses of covariance (ANCOVA); *F*(156,4) = 5.6665, *P* = 0.0010, *n* = 160]. SN MRI contrast and volume were not associated with Aß42/40, ptau and total tau CSF levels (all *P* > 0.1) or hippocampal novelty activation (all *P* > 0.1). Moreover, SN volume was positively associated with recognition memory (*R*^2^ = 0.07, *P* < 0.001, *n* = 159), global cognition (*R*^2^= 0.38, *P* < 0.0001, *n* = 160) and years of education (*R*^2^ = 0.03, *P* = 0.036, *n* = 160). Our study emphasizes the potential of using *in vivo* SN MRI markers to study the impact of SN degeneration on general cognitive impairment and recognition memory in an Alzheimer’s disease dementia cohort. Our results motivate future longitudinal studies to explore how SN volume and SN contrast change with disease progression, how these are differentially associated with cognitive decline, and how SN volume and SN contrast might be related to other dopamine-dependent cognitive functions and dysfunctions.

## Teaser

Krohn *et al*. examined Substantia Nigra MRI contrast, volume and cognition in a cohort with Alzheimer’s disease cohort. Volumetric diagnostic group differences and the significant link between Substantia Nigra (SN) volume, memory and cognition demonstrate SN volume’s value for studying the dopaminergic system in Alzheimer’s disease.

## Graphical Abstract

**Graphical Abstract:**
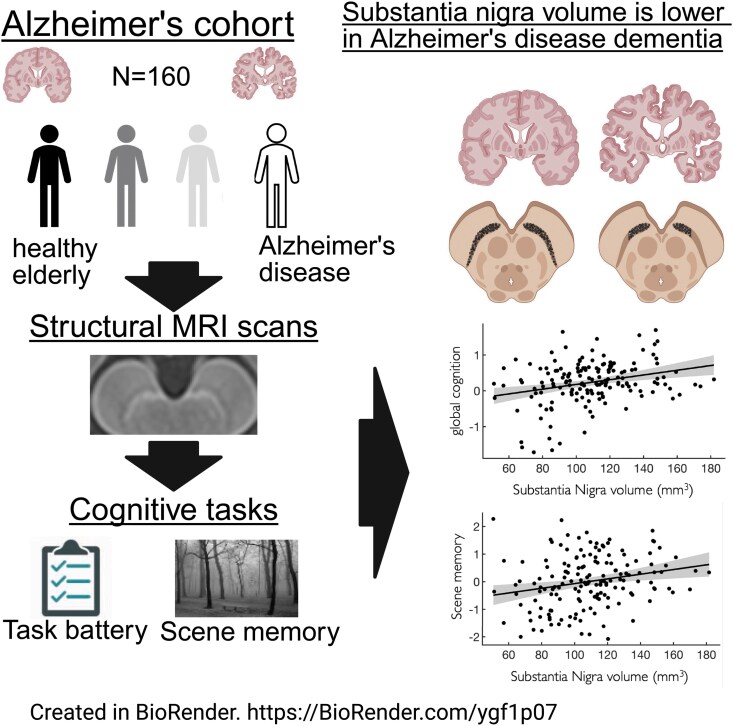

## Introduction

The Substantia Nigra (SN), a dopaminergic midbrain region is involved in various functions, including movement initiation,^1^ executive function,^2^ working memory,^3^ long-term memory^4^ and novelty processing.^5^ Indeed, the SN and the surrounding ventral tegmental area (VTA) cells show increased fMRI and cellular activity when individuals are presented with novel stimuli.^5-7^ Dopaminergic hippocampal novelty signals facilitate information encoding into long-term memory.^8-11^ Therefore, the SN might be linked to novelty-related memory formation.

The SN contains high levels of neuromelanin (NM),^12^ a dark, iron-rich pigment. In SN-sensitive MRI, regions rich in NM,^4,13-16^ a dark iron-rich^17^ pigment,^17^ appear hyperintense.^13^ Different SN MRI contrasts and SN volume have been calculated from SN-sensitive MRI. SN MRI contrast measures have been linked to memory and cognitive control in older adults.^4,18,19^ Reduced post-mortem SN volume^20^ and NM concentration in Parkinson’s Disease^21^ compared to HC is paralleled by lower SN-MRI-derived volumetric^16^ and contrast.^22^ Furthermore, SN measures are sensitive to working memory decline^15^ in individuals with Parkinson’s disease, emphasizing the usability of SN-sensitive MRI for non-invasive SN health assessment.

While the role of the SN has been extensively studied in ageing, its role in Alzheimer’s disease remains underexplored. Alzheimer’s disease is a neurodegenerative disease characterized by the presence of pathological β-amyloid and hyperphosphorylated tau.^23,24^ A meta-analysis showed lower dopamine levels and D1 and D2 receptor density^23^ in individuals with Alzheimer’s disease dementia compared to healthy controls (HC),^25^ indicating that the dopaminergic system is affected in Alzheimer’s disease. SN volume and dopaminergic functions are impacted by Alzheimer’s disease: Post-mortem cases indicate that SN volumetric reduction in clinically defined dementia resulting from Alzheimer’s disease is mild on average,^20,26,27^ but more pronounced than in healthy elderly.^26^ Post-mortem studies indicate that SN volume in Alzheimer’s disease dementia varies significantly among individuals—some show little to no degeneration.^28^ In contrast, a subset of individuals demonstrates degeneration similar to that of individuals with Parkinson's disease.^20,25,28,29^ and more volumetric cell loss with more advanced Alzheimer’s disease dementia status.^29^ Moreover, insoluble tau and amyloid pathology have been shown post-mortem in SN cells in 60–91% of cases with Alzheimer’s disease dementia,^26,27,29-32^ and insoluble tau tangle levels are higher in post-mortem SN sections of individuals at advanced stages of Alzheimer’s disease.^29^

Individuals with Alzheimer’s disease dementia show both behavioral^33^ and hippocampal activation deficits during novelty processing^34^ and do not show enhanced memory for novel compared to familiar events.^35,36^ While post-mortem studies indicate that the SN is affected in Alzheimer’s disease dementia, to our knowledge, no studies have investigated how SN degeneration is related to novelty and long-term memory deficits in Alzheimer’s disease dementia. Here, we evaluate the role of the SN as a proxy for dopaminergic function in Alzheimer’s disease dementia, utilizing data from the prospective DZNE—Longitudinal Cognitive Impairment and Dementia Study (DELCODE). Specifically, we examine the relationship between *in vivo* SN MRI measures, novelty processing and recognition memory by combining SN-sensitive MRI and task fMRI in individuals across the Alzheimer’s disease dementia continuum. We also explore how SN MRI contrast and volume are influenced by Alzheimer’s disease pathology in a subset of individuals with available CSF biomarker levels. Finally, we analyse the association between SN MRI markers, novelty activation and recognition memory in a subset of individuals with CSF AD biomarker levels.

## Materials and methods

### Subjects

We here analyse a subset of the multicentre DELCODE study^37^ containing 160 subjects (72 men, 69 ± 6 years) comprising 79 HC, 54 participants with subjective cognitive decline (SCD), 17 participants with mild cognitive impairment (MCI) and 10 individuals diagnosed with Alzheimer’s disease dementia (for participant statistics see Table 1). All participants were older than 60, fluent in German and provided informed consent. HCs were recruited through local advertisements, while SCDs, MCIs and individuals with Alzheimer’s disease dementia were recruited through (self-) referrals. All subjects performing within 1.5 SD on the age-, sex- and years of education-adjusted CERAD (Consortium to Establish a Registry of Alzheimer's Disease) and not complaining of memory problems were considered HC. Subjects performing within the age- sex- and years of education-adjusted healthy range of 1.5 SD on CERAD but complaining of memory problems to physicians in memory clinics were regarded as individuals with SCD per recent guidelines.^38^ Subjects performing below 1.5 SD on the age-, sex- and years of education-adjusted delayed recall CERAD task and complaining of memory problems to physicians in memory clinics and had intact daily functioning were classified as individuals with MCI per recent guidelines.^39^ Finally, subjects who fulfilled the clinical criteria for Alzheimer’s disease dementia according to current standards^40^ were classified as individuals with Alzheimer’s disease dementia. Individuals scoring below 18 points on the MMSE were excluded,^36^ according to previously published recommendations.^41^ For exclusion criteria and further DELCODE study details, see Jessen *et al*.^37^

**Table 1:** Overview of the demographics, cognitive test results and AD CSF pathology load in the sample analysed in this work

	HC	SCD	MCI	ADD
**Whole dataset**				
*N*	79	54	17	10
Age	67.2 (5.1)	70.5 (6.1)**	71.72 (7)*	72.1 *(6.6)
Sex (M:F)	28:51	30:24	11:7	3:7
SN volume [mm^3^]	109.3 (25.6)	114 (27.5)	102.3 (27.3)	83.18 (17)**
Years of education	14.8 (2.67)	14.95 (3.04)	13.53 (2.92)	12.8 (2.7)
MMSE	29.35 (0.95)	29.02 (1.11)	27.82 (1.42)***	22.9 (2.92)***
TIV [ml]	1353.61 (205.53)	1405.38 (223.31)	1472 (259.05)	1445.56 (170.08)
**Subset with available CSF data**				
*N*	30	20	14	7
Ttau (pg/ml)	322.1 (116.3)	346.3 (178.5)	614.1 (291.1)**	883.7 (502.2)***
Ptau (pg/ml)	45.6 (13.9)	48.4	72.3 (32.9)*	106.6 (69.2)***
Aβ42/40 ratio	0.1 (0.02)	0.1 (0.002)	0.006 (0.003)***	0.005 (0.02)***
Aβ42/Ptau181 ratio	18.5 (5.5)	17.0 (4.4)	10.3 (8.0)**	5.8 (6.0)***

Stars denote the *P* value of a two-sided *t*-test compared with HC: *P < 0.05, **P < 0.01, ***P < 0.0001, HC, healthy control; SCD, subjective cognitive decline; MCI, mild cognitive impairment; ADD, Alzheimer’s disease dementia, the numbers describe the blinded scanner ID.

One-way ANOVAs assessing diagnostic group differences between all covariates, cognitive factors and CSF biomarkers showed that in our sample, individuals with ADD were older [*F*(3156) = 6.04, *P* < 0.0001] (Table 1), were more likely to be male [*F*(3156) = 3.07, *P* = 0.03], had abnormal levels of amyloid [*F*(3156) = 11.87, *P* < 0.0001], total tau [*F*(3156) = 14.81, *P* < 0.0001] and phosphotau [*F*(3156) = 10.81, *P* < 0.0001] levels, had lower scores in recognition memory [*F*(3156) = 19.52, *P* < 0.0001], had lower scores in global cognition [*F*(3156) = 59.72, *P* < 0.0001] and had lower factor scores: working memory abilities [*F*(3156) = 31.14, *P* < 0.001], language abilities [*F*(3156) = 69.56, *P* < 0.001], memory abilities [*F*(3156) = 85.06, *P* < 0.0001], executive function [*F*(3156) = 44.93, *P* < 0.0001] and visuospatial abilities [*F*(3156) = 28.24, *P* < 0.0001]. All tested diagnostic group differences are listed in Supplementary Table S2.

The Institutional Review Boards of all participating study centres of the DZNE approved the study. All participants provided written informed consent prior to their inclusion in the study. DELCODE is retrospectively registered at the German Clinical Trials Register (DRKS00007966) (04/05/2015). Data handling and quality control are reported elsewhere.^37^

### Structural MRI scans

MRI data for the present study were acquired using Siemens scanners (Verio and Skyra systems) at four study centres (blinded site numbers 5, 10, 13 and 18). For the analyses reported here, T1-weighted MP2RAGE [3D GRAPPA PAT 2, 1 mm^3^ isotropic, 256 × 256 px, 192 slices, sagittal, 5 min, repetition time (TR) 2500 ms, echo time (TE) 4.33 ms, inversion time (TI) 110 ms, flip angle (FA) 7°] images were used. Whole brain T1-weighted fast low-angle shot (FLASH) SN-sensitive MRI images were acquired using the following parameters: 0.75 × 0.75 × 0.75 mm^3^ voxel size, 320 × 320 × 192 matrix, 5.56 ms echo time, 20 ms repetition time, 23° flip angle, 130 Hz/pixel bandwidth, 7/8 partial Fourier and 13:50 min scan duration, as previously reported.^42^

### Novelty task

**Figure 1:**
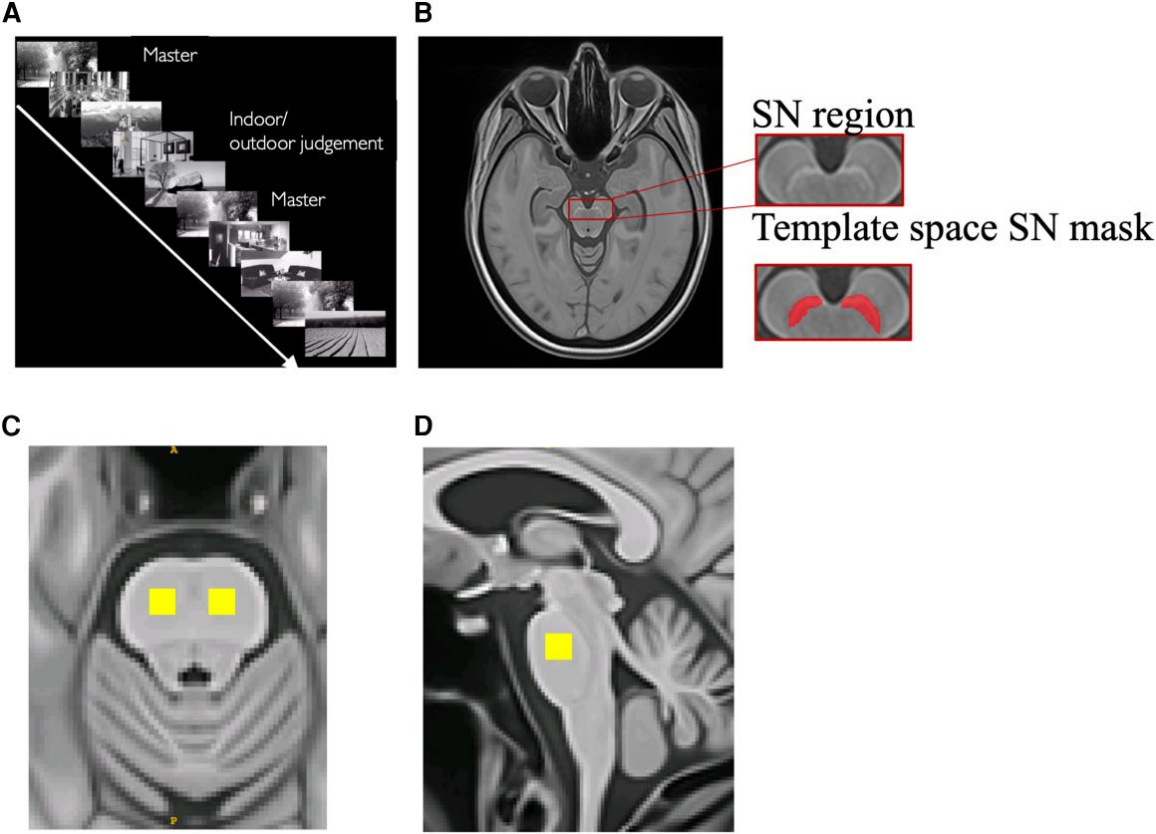
**FADE task instruction and template space SN and pons segmentations**. (**A**) Subjects were presented with 88 novel scenes interleaved with two pre-familiarized scenes, and they were instructed to label the images as inside or outside. Seventy minutes later, they rated the 88 previously presented images and 44 novel images by their familiarity in front of a computer. (**B**) We generated a template image from 188 upsampled FLASH images using ANTs, from which the SN was manually segmented and morphed into the upsampled subject space and then corrected manually. (**C**) A pons mask (in yellow) was segmented in template space and morphed into native space for SN intensity normalization. (**D**) Same as (**C**) but in coronal plane.

Participants first performed a previously published^43^ and adapted^44^ fMRI novelty and a recognition memory test. During the novelty part (Fig. 1A), inside the fMRI scanner, participants labelled two pre-familiarized images of scenes each 44 times interleaved with 88 novel images as indoor or outdoor scenes. Stimuli were displayed as 8-bit greyscale images scaled to a 1250 × 750 pixel resolution and matched for luminance; the viewing horizontal half-angle was 10.05° (‘Presentation’ software by Neurobehavioral Systems Inc.). All participants underwent vision correction using MR-compatible goggles, following standard operating procedures. All sites used the same 30′ MR-compatible LCD screen, matched for distance, luminance, colour and contrast across sites, and all sites used the same response buttons. Stimuli were shown for 2500 ms each. fMRI images were recorded using the following parameters: 3.5 mm isotropic resolution, 64 × 64 px, 47 slices, oblique axial/AC-PC aligned, 9 min, TR 2580 ms, TE 30 ms, FA 80, 206 volumes. The novelty part took around 11 min. The recognition memory part was then performed in front of a PC 70 min after the fMRI task: participants rated the 88 recently presented scenes but not the prefamiliarized scenes and 40 additional images on a 5-scale familiarity scale, with 1 indicating they were sure they did not see the image, 3 indicating they are not sure if saw the image and 5 indicating they were sure they previously saw the image.

### fMRI preprocessing

First-level general linear models were calculated after preprocessing [slice time correction, unwarping, realignment and spatial smoothing (isotropic Gaussian kernel of FWHM 6 × 6 × 6 mm)] using Statistical Parametric Mapping (SPM, Version 12) in native space (including six motion regressors from the realignment process). We used a haemodynamic response function with a 128-s high-pass filter, no global scaling and no serial correlations modelled. The novelty contrast was calculated as the difference between activation in response to novel and activation in response to familiar images.

### Group-level analyses

Using the Advanced Normalization Tools toolbox,^45^ a study-specific group template was calculated from T1-weighted MPRAGE images. Four rigid-then-affine iterations and six runs of a nonlinear multiresolution routine ensured stable convergence (three resolutions, a maximum of 90 iterations, and a template update step size of 0.1 mm). Individual native space novelty contrast fMRI statistical images (moving images) were warped to the group template space (fixed image) using the Advanced Normalization Tools toolbox function antsregistrationSyn. Voxelwise mass-univariate associations between structural SN MRI measures and hippocampal fMRI novelty activation were later performed in this space. Grey matter volume was calculated using the CAT toolbox.^46^ Freesurfer 6.0 was used to segment a hippocampal mask image (6896 voxels) from the template based on the Desikan–Killiany atlas.^47^ The FLASH images were upsampled using the MATLAB interpn function with a sinc interpolation method and an upsampling factor of two. The resulting image was a semiquantitative image optimized for the visibility of the SN at 0.375 mm isotropic resolution. Using the Advanced Normalization Tools toolbox, a study-specific template was generated from the upsampled FLASH images (Fig. 1B). The SN and a pons reference region (Fig. 1C and D) were manually segmented from the upsampled template using ITKSnap. The segmented SN and pons in that template space image were morphed back into the upsampled native space of all subjects. In the upsampled native space, experienced SN segmenters (M.S., M.B.) corrected and checked all masks using ITKSnap. The SN MRI contrast was calculated as the ratio between the median intensity (int) inside the SN mask and the median int in the pons reference region, as previously described^42^ using the following formula:


$$\frac{Int_{SN}-Int_{Pons}}{Int_{Pons}}$$

SN volume was calculated using FSLmaths as the number of non-zero mask voxels multiplied by the voxel volume. We use bilateral values, which are the sum of the left and right. To account for site differences, we harmonized the MRI factors across sites using the ComBAT toolbox.^48^ The harmonization allows for adjusting site difference averages. Preserving diagnostic group variance allowed for maintaining diagnostic group differences while reducing site effects. MB and MS generated manual and semiautomatic segmentations of FLASH scans from 24 younger individuals and 43 elderly healthy individuals from a separate, independent cohort as previously described.^42^ When comparing volume and contrast across all individuals, the volumetric dice scores for SN volume were 0.66 (SD: 0.15732), and the ICC for SN MRI contrast was 0.992. However, when comparing only the elderly individuals, the volumetric dice was 0.7432 (SD: 0.092) and the contrast ICC was 0.992. This indicates high agreement in SN contrast and satisfactory volumetric agreement.

### Cognitive measures

All subjects were tested using the CERAD battery^49^ and the neuropsychological^49^ assessment battery,^50^ which comprises the mini-mental state exam, ADAS Cog, the Free and Cued Selective Reminding Test^51^ which includes a serial subtraction task, Wechsler Memory Scale revised version^52^ Logical Memory (Story A) and Digit Span,^53^ 2 semantic fluency tasks (animals and groceries),^54^ the Boston Naming Test (15-item short version analogue to the CERAD battery),^55^ supplemented by 5 infrequent items from the extended version^56^ the oral form of the Symbol-Digit-Modalities Test (including a subsequent free recall of symbols and symbol-digit pairings).^56^ Trail Making Test Parts A and,^57^ Clock Drawing and Clock Copying^58^ and a recall task of previously copied figures (as in the CERAD test battery).^55^ In addition to these established tests, two newly developed computerized tests were implemented: the Face Name Associative Recognition Test^59^ and a Flanker task^60^ to assess executive attention control. Confirmatory factor analysis was then used to derive five cognitive domain scores: learning, memory, language, executive function, visuospatial abilities and working memory. A global cognitive score was calculated as previously described from the five cognitive scores.^61^ The sample used to generate the global cognitive score contained more subjects than the sample analysed in this study.

### CSF measures

For 71 participants in our subset, CSF levels of Alzheimer’s disease biomarkers, including the Aβ42/40 ratio, p-tau-181 and t-tau, were measured. In adherence with recent guidelines in the ATN framework,^24^ we thus estimated the Aß42/40 ratio, tau pathology (p-tau-181) and neurodegeneration (total tau). The CSF levels were measured centrally in Bonn using commercially available kits according to vendor specifications: V-PLEX Ab Peptide Panel 1 (6E10) Kit (K15200E) and V-PLEX Human Total Tau Kit (K151LAE) (Meso, MD, USA) and Innotest Phospho-Tau(181P) (81581; Fujirebio Germany GmbH, Hannover, Germany) as described previously.^37^ We binarized the subjects into pathology positive/negative (i.e. Aβ42/40 ratio smaller than 0.08 as Aβ42/40 positive, subjects with total tau levels greater than 510.9 pg/ml as tau positive and subjects with levels greater than 73.65 pg/ml as phosphotau 181 positive) and vice versa^61^ using study-specific cut-offs.^62^

### Statistical analysis

Analyses were performed in MATLAB 2022a (Natick, Massachusetts). We used linear models to investigate the relationship between SN MRI markers, CSF biomarker levels and recognition memory. Calculating analyses of covariance (ANCOVAs) using the Python statsmodel module,^63^ we also assessed diagnostic group differences in SN volume and SN MRI contrast while accounting for age, sex, years of education and TIV. We used the Statistical Parametric Mapping Toolbox 12 Revision 7771 (SPM) to calculate mass-univariate models to assess the relationship between SN MRI contrast and volume and hippocampal novelty contrast, applying family-wise error (FWE) cluster-level correction for multiple comparisons. We limited our voxelwise analysis to the hippocampal mask defined in 2.6. In all analyses, we used age, TIV, years of education and sex as covariates. Linear associations were plotted using the Gramm toolbox for MATLAB,^64^ and group comparisons were drawn using the Raincloud toolbox. To account for diagnostic group differences, we also repeated every analysis yielding a significant association between the outcome measure and SN MRI factors, using the same covariates and correcting for overall grey matter volume, hippocampal volume and diagnostic group in separate models. We calculated Hedge’s g^65^ to calculate the effect sizes of the diagnostic group differences. Additionally, we corrected all associations between SN MRI measures and demographics to account for multiple comparisons. Nested analyses, such as additional sub-analyses that additionally corrected for diagnostic group and hippocampal volume, as well as analyses that included only subgroups of subjects, were excluded from multiple comparisons. We report the uncorrected *P*-value as *P* and the corrected *P*-value as *q*. Given their statistical independence, we considered the association between each MRI marker and cognitive markers as independent hypotheses and performed separate multiple comparisons for SN contrast and SN volume.

## Results

Associations between SN volume and SN contrast were not significant after correcting for age, sex, years of education and TIV (*R*^2^ = 0.03, *P* = 0.2203, *n* = 160), indicating that the MRI measures are not statistically associated in our sample. Two-sided *t*-tests between the left and right SN volume and SN MRI contrast revealed a significantly higher SN volume (*P* < 0.001) and significantly higher SN MRI contrast (*P* = 0.0092) in the right compared to the left SN hemispheres. Two-sided *t*-test revealed no sex differences in SN contrast [*t*(158) = 0.47, *P* = 0.638, *q* = 0.893] and SN volume [*t*(158) = −1.3, *P* = 0.194, *q* = 0.279]. All tested associations are listed in Supplementary Table S1. Furthermore, we found an association between SN volume and years of education (*R*^2^ = 0.032, *P* = 0.036, *q* = 0.063, *n* = 160) and between SN contrast and age (*R*^2^ = 0.078, *P* = 0.006, *q* = 0.084, *n* = 160). However, these results did not survive multiple comparisons. Most individuals were assessed at Site ID 10 (83 individuals), followed by Site ID 13 (54), Site ID 5 (22) and Site ID 18 (1).

### SN volume is lower in Alzheimer’s disease dementia but is not associated with CSF Aß42/40 and tau levels

**Figure 2:**
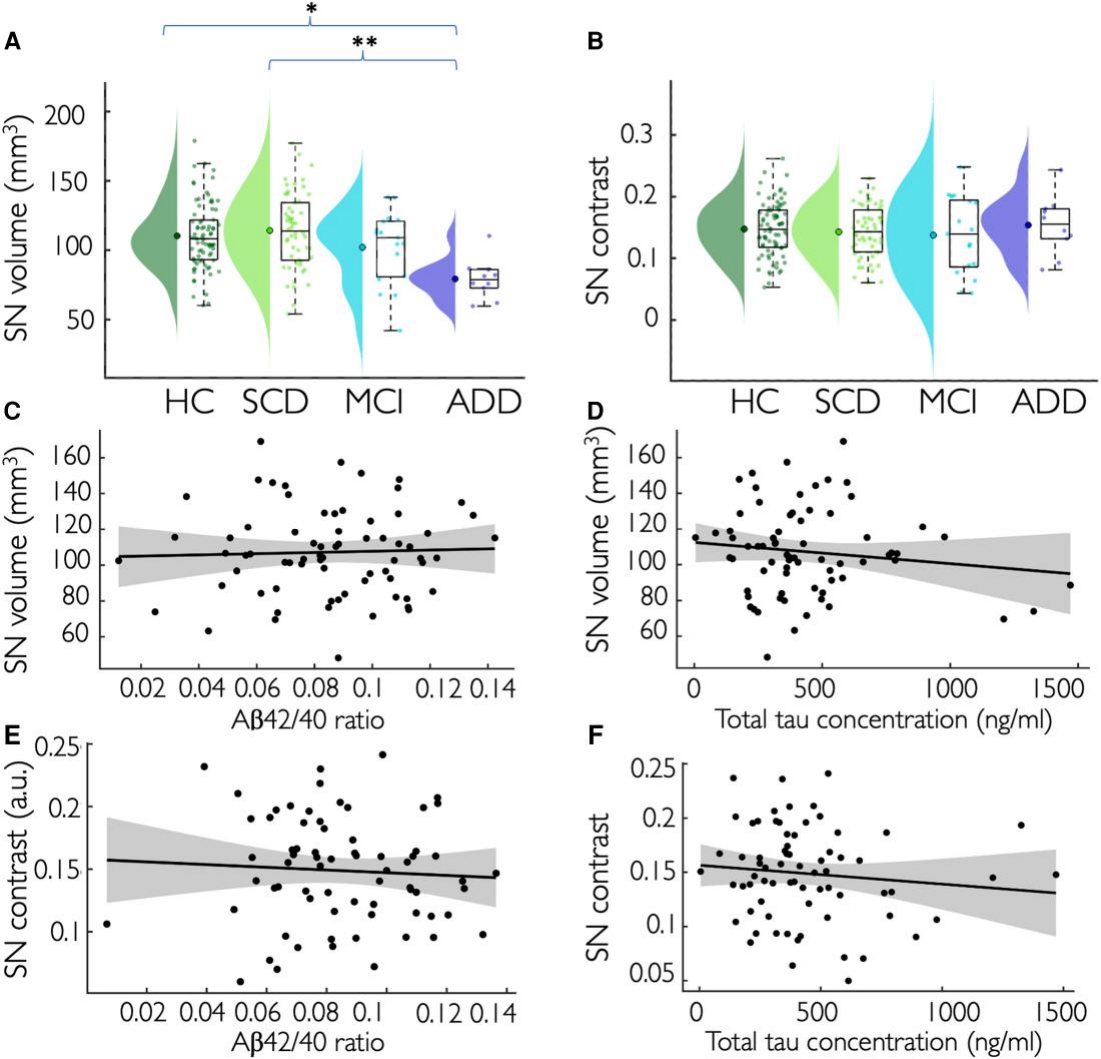
**SN volume is lower in individuals with Alzheimer's disease dementia but is not associated with Alzheimer's disease pathology**. (**A**) An ANCOVA comparing Substantia Nigra (SN) volume across diagnostic groups [*F*(152,8) = 5.6665, *P* = 0.0010, *n* = 160] and a subsequent *post hoc* test revealed a significantly lower SN volume in individuals with Alzheimer's disease dementia compared with HC (*P* = 0.016, 23% average volume difference, Hedges’ *g* = 1.4) and SCD (*P* = 0.004, 28% average volume difference, Hedges’ *g* = 1.3). (**B**) An ANCOVA between SN MRI contrast and diagnostic groups revealed no significant difference [*F*(152,8) = 3.5166, *P* = 0.223, *n* = 160]. (**C–F**) Linear models revealed no significant association between SN MRI measures and Aβ42/40 ratio (**C, E**) or total tau (**D, F**). Legends for (**A + B**): The patch next to the datapoint denotes probability density. The point on the patch denotes the mean. The box edges in the group comparisons box plot denote 1 IQR, while the whiskers denote 1.5 IQR. HC, healthy control subjects; SCD, subjective cognitive decline; MCI, mild cognitive impairment; ADD, individuals with Alzheimer‘s disease dementia. **C–F**, The line denotes the best fit, and the shaded area indicates the 95% confidence interval. HC, healthy control subjects; SCD, individuals with subjective cognitive decline; MCI, individuals with mild cognitive impairment; ADD, individuals with mild Alzheimer‘s disease dementia.

Correcting for age, sex, years of education and TIV, a significant difference in SN volume between diagnostic groups was observed [ANCOVA; *F*(152,8) = 5.6665, *P* = 0.0010, *n* = 160, Fig. 2A]. Subsequent *post hoc* comparisons revealed that SN volume was lower in individuals with Alzheimer’s disease dementia compared to HC (*P* = 0.016, 23% average volume difference, Hedges’ *g* = 1.4) and compared to individuals with SCD (*P* = 0.004, 28% average volume difference, Hedges’ *g* = 1.3). This indicates that in our dataset, a significantly lower SN volume compared to HC can be detected in individuals with Alzheimer’s disease dementia, but not in MCI. No significant difference in SN MRI contrast between diagnostic groups was observed [*F*(152,8) = 3.5166, *P* = 0.223, *n* = 160, Fig. 2B]. Separate linear models assessing the relationship between the SN MRI markers and all CSF biomarker levels while correcting for age, sex, years of education and TIV revealed no significant effect for the MRI markers (all *P* > 0.1, Fig. 2C–E).

### Higher SN volume predicts higher recognition memory and a higher global cognitive score

**Figure 3:**
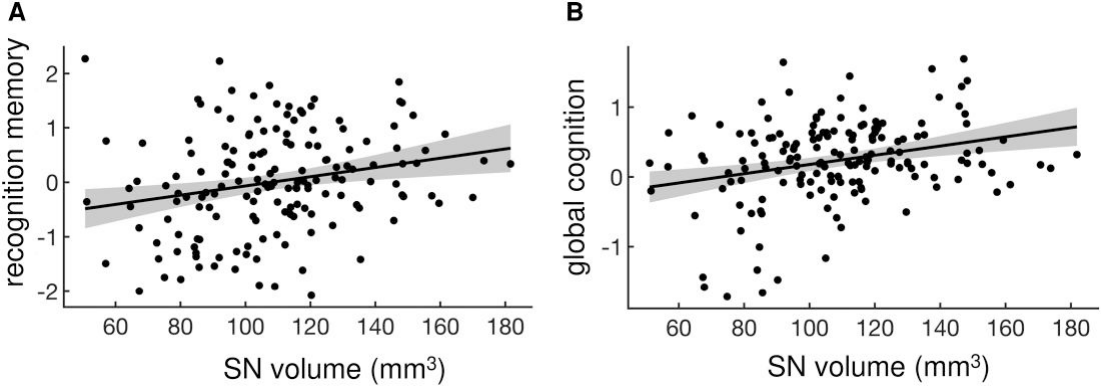
**Substantia Nigra (SN) volume is associated with recognition memory and overall cognition**. Separate linear models regressing SN volume and each recognition memory and global cognition were significant. (**A**) In a linear model correcting for age, sex, years of education and TIV, higher SN volume predicted better recognition memory (*R*^2^ = 0.071, *P* = 0.004, *q* = 0.011, *n* = 159). (**B**) In a linear model correcting for age, sex, years of education and TIV, higher SN volume predicted better global cognition (*R*^2^ = 0.096, *P* < 0.001, *q* < 0.001, *n* = 160). In both panels, the line denotes a linear fit, while the shaded area denotes 1 STD.

We associated SN volume and SN contrast with recognition memory while accounting for age, sex, years of education and TIV and demonstrated a positive association between SN volume and recognition memory (*R*^2^ = 0.071, *P* = 0.004, *q* = 0.011, *n* = 159, Fig. 3A). This association remained significant after correcting for hippocampal volume (*R*^2^ = 0.065, *P* = 0.004, *n* = 159) and overall grey matter volume (*R*^2^ = 0.107, *P* = 0.016, *n* = 159) but not after correcting for diagnostic group (*P* = 0.11). To determine if SN volume is related to overall cognitive function, we conducted an additional analysis linking SN volume and contrast with a global cognitive score aggregated across five cognitive domains (visuospatial abilities, memory, working memory, language and executive function), while also correcting for years of education, age, sex and TIV. We showed a significant association between SN volume and global cognition (*R*^2^ = 0.096, *P* < 0.001, *q* < 0.001, *n* = 160, Fig. 3B). This association remained significant after correcting for hippocampal volume (*R*^2^ = 0.091, *P* < 0.001 *n* = 160) and total grey matter volume (*R*^2^ = 0.112, *P* = 0.007, *n* = 160) but and diagnostic group (*P* = 0.0.71). In addition to finding a link between SN volume and global cognition, we also observed significant correlations between SN volume and latent factors reflecting working memory (*R*^2^ = 0.09, *P* = 0.001, *q* = 0.004, *n* = 160, Supplementary Figure S1A), visual–spatial abilities (*R*^2^ = 0.062, *P* = 0.009, *q* = 0.018, *n* = 160, Supplementary Figure S1B), memory (*R*^2^ = 0.066, *P* = 0.006, *q* = 0.014, *n* = 160, Supplementary Figure S1C), executive function (*R*^2^ = 0.098, *P* < 0.001, *q* < 0.001, *n* = 160, Supplementary Figure S1D) and language (*R*^2^ = 0.1, *P* < 0.001, *q* < 0.001, *n* = 160, Supplementary Figure S1E). These associations held after correcting for hippocampal volume and total grey matter volume and diagnostic group (Supplementary Table S1). These results suggest that greater SN volume is associated with enhanced recognition memory and cognitive abilities across multiple domains. These associations were not statistically linked to hippocampal atrophy in individuals across the Alzheimer’s disease dementia continuum. We associated each cognitive factor with an SN volume by diagnostic group interaction (Supplementary Figure S2), correcting for age, sex, years of education and TIV to test for slope differences between groups. For dprime, the SN volume, a diagnostic group interaction, or the diagnostic group term was significant. However, for the global cognitive score and the factor scores, only the global cognitive score or the factor scores, respectively, were significant. Overall, these results indicate that diagnostic group interactions might have driven the association between SN volume and recognition memory. However, given the low sample size of individuals with MCI and ADD, this association should be interpreted cautiously.

Finally, we also tested for group differences in all cognitive markers assessed in this manuscript using ANOVAs and found diagnostic group differences across all markers (Fig. 1 and Supplementary Figure S2).

A linear model assessing the relationship between SN volume and age, sex, years of education, TIV, recognition memory and global cognition (*R*^2^ = 0.115, *n* = 160) was significant for global cognition (*P* = 0.01) but not recognition memory (*P* = 0.23), indicating that recognition memory and global cognition are not independently associated with SN volume. An association between recognition memory and global cognition (*R*^2^ = 0.56, *P* < 0.0001, *n* = 160), accounting for age, sex, years of education and TIV, was significant. However, given that memory retrieval is crucial for overall cognitive function, one can expect an overlap in variance between recognition memory and overall cognitive function. We then tested for unique associations between SN volume and recognition memory, the global cognitive score, and the factor scores by calculating the linear model SN volume ∼ recognition memory + global cognitive score + working memory + language + visuospatial abilities + executive function + age + sex + years of education + TIV. The lack of any significant factor showed that SN volume is not uniquely associated with any cognitive measure, pointing towards an association between SN volume and general cognitive abilities rather than a specific domain.

An association between hippocampal novelty activation, SN contrast and SN volume revealed no significant voxel, suggesting that the SN MRI factors are not statistically associated with hippocampal novelty activation in this sample.

## Discussion

Using *in vivo* structural MRI measures of the SN (SN volume and MRI contrast), we assessed how the SN is affected in Alzheimer’s disease dementia and how the SN is related to hippocampal activity during a novelty task, subsequent recognition memory and general cognition. In a subset of individuals with CSF biomarker data, we also assessed how SN degeneration is related to Alzheimer’s disease pathology. We did not find a statistically significant association between SN contrast and SN volume, indicating that they were not significantly correlated in our dataset. We also revealed that SN volume is lower in individuals with Alzheimer’s disease dementia than in HC and in individuals with SCD. However, SN volume did not differ between HC and MCI and was not associated with CSF biomarkers of Alzheimer’s disease pathology. While higher SN volume was associated with better recognition memory and better overall cognitive function, higher SN MRI contrast was associated with higher age.

Previous studies have estimated SN MRI contrast and SN volume measures, but did not report their relationship.^66-69^ Here, we found no association between SN MRI contrast and SN volume.

While this study demonstrated only the statistical independence of SN contrast and SN volume, SN MRI contrast and SN volume likely represent distinct constructs. SN contrast may indicate the strength of the contrast mechanism that causes voxel hyperintensity in the SN region on SN-sensitive MRI. In contrast, SN volume could reflect the volumetric extent of the contrast mechanism. The mechanism is likely multivariate, consisting of NM-linked mechanisms, such as paramagnetic T1 shortening,^70-73^ and NM-independent mechanisms, including magnetization transfer^71,72^ and a lower macromolecular fraction,^73^ as well as a high intracellular water density.^74-76^ Given that dopaminergic neurons are large^77^ and are the most abundant neuron type in the SN^78^ is plausible that dopaminergic neurons primarily drive the contrast mechanism. Although not systematically analysed, SN volume could reflect the overall structural integrity and completeness of dopaminergic neurons, while SN contrast may indicate the average cellular health.

Our study revealed that MRI-derived SN volume was 25% lower in individuals with Alzheimer’s disease dementia than in HCs. This difference aligns with previous post-mortem studies, which report varying degrees of SN volume reduction, ranging from 10% to 40%.^20,79,80^ One study showed no difference in SN volume,^28^ contradicting our results. Moreover, we found no significant volumetric difference in the SN between MCI and SCD compared with HC, indicating that SN volume differences can only be detected at the stage of Alzheimer’s disease dementia. However, given the small number of individuals with MCI and Alzheimer’s disease dementia, these results should be considered preliminary.

Interestingly, studies assessing group differences in SN-sensitive MRI contrast using TSE MRI sequences^81,82^ also showed no significant difference in SN MRI contrast between HC and individuals with Alzheimer’s disease dementia, in line with our results. As we do not find an association between SN volume and SN contrast, these cross-sectional findings suggest that SN atrophy may be more closely linked to cognitive decline than microstructural changes detectable through MRI contrast (likely reflecting neuromelanin, iron content, or free water alterations). The lack of association between SN MRI contrast and cognition may also reflect the limited sensitivity of our MRI contrast to microstructural differences in ADD. Since our subset contained only 10 individuals with Alzheimer’s disease dementia and since variability in SN volume loss is high,^20^ future studies should reproduce these results in a larger sample size of individuals with MCI and Alzheimer’s disease dementia.

We showed no significant association between SN MRI contrast and SN volume and Alzheimer’s disease pathology in a subset of individuals with CSF biomarker status. Several post-mortem studies also showed no association between SN cell loss and tau or amyloid pathology.^27,30^ While one study^29^ showed that higher tau plaque concentrations in the SN coincide with lower SN volume in individuals at more advanced stages of Alzheimer’s disease, the authors did not show a direct association between SN degeneration and Alzheimer’s disease pathology. Our results are not in direct contradiction with these findings. Future studies using larger samples are still needed to confirm the relationships between SN degeneration and Alzheimer’s disease pathology. Previous reports also show that alpha-synuclein is related to SN degeneration in Alzheimer’s disease dementia. In one study, alpha-synuclein was moderately associated with SN loss in Alzheimer’s disease dementia.^30^ Alpha-synuclein is toxic to the SN pars compacta,^83^ notably in Parkinson's disease.^84^ Additionally, elevated alpha-synuclein levels have consistently been demonstrated in individuals with MCI and Alzheimer’s disease dementia.^29,85-88^ Therefore, the volumetric SN difference observed in our study may also be related to elevated alpha-synuclein levels. While alpha-synuclein was not assessed in the DELCODE study, it will be important in future studies to investigate the extent to which alpha-synuclein pathology is related to SN degeneration in Alzheimer’s disease dementia.

Higher SN MRI contrast,^4^ higher SN/VTA MRI activity,^7,89-94^ and higher cellular SN activity^5^ predict better long-term memory. We extend these previous findings by showing that SN volume is associated with recognition memory in individuals spanning the Alzheimer’s disease continuum. A lower novelty signal from the SN during the presentation of previously presented images during the retrieval phase of the novelty task could help improve participants’ recognition memory precision. We also expand previous findings showing that SN volume is linked to broader cognitive functions, evident by the significant associations with several cognitive factor scores spanning five cognitive domains and to global cognition.^98^ These results indicate an overall cognitive function of the SN in line with previously shown basal ganglia involvement in working memory gating.^95^ However, the results also showed that the associations between SN volume and cognition were not specific to recognition memory but rather point towards a broader relationship between SN volume and general cognitive functions, possibly through its role in working memory^96^ and recognition memory networks.^10^ Future studies should investigate the direct relationship between SN volume and other dopaminergic functions, such as reward^97^ and examine the temporal and causal relationship between SN volume decline, recognition memory decline and global cognition.

Furthermore, after correcting for an SN volume by diagnostic group interaction, the association between SN volume and recognition became non-significant. This suggests that diagnostic group differences may drive the association between SN volume and recognition memory. However, given the low number of subjects with MCI and ADD, these results should be interpreted cautiously as the statistical power is too low to assess within-group associations. Future studies with larger sample sizes of MCI and ADD should assess the link between SN volume, diagnostic group and cognition.

It is important to note several limitations of our study. The number of individuals with Alzheimer’s disease dementia was low. Thus, the results presented here need to be replicated in larger samples. The contribution of VTA degeneration, another source of dopamine in the brain that contributes to novelty and memory deficits in Alzheimer’s disease dementia,^98^ was not disentangled from the role of the SN. It might explain the lack of association between SN MRI factors and the novelty response. Our SN segmentations were performed semi-automatically, which may have introduced bias and overestimated the SN. Finally, our study was cross-sectional. Longitudinal follow-up data, once available, will enable us to investigate the relationship between SN degeneration and cognitive function over time.

Future studies should attempt to replicate our results in larger cohorts of individuals with Alzheimer’s disease dementia with Alzheimer’s disease pathology and CSF levels. They should also assess how SN contrast and SN volume are structurally and functionally related. Further investigation is required to assess whether SN volume, as previously observed for SN MRI contrast, is related to other dopamine-dependent functions. Finally, further research is required to systematically assess the exact cellular mechanisms underlying SN degeneration in Alzheimer’s disease dementia, for instance, the contribution of alpha-synuclein.

In conclusion, this work demonstrates that SN volume is sensitive to degeneration in Alzheimer’s disease and is also associated with recognition memory and global cognitive function. We did not find any association between SN MRI markers and Alzheimer’s disease pathology, and further research is required to determine if SN degeneration is related to additional pathology, such as alpha-synuclein.

## Supplementary Material

fcag238_Supplementary_Data

## Data Availability

Data, study protocol and biomaterials can be shared with partners based on individual data and biomaterial transfer agreements. Scripts used for data analyses of this manuscript can be found here: https://github.com/frikro/SN-Novelty-Brain-Comm.
