# Supplementary material for: Substantia Nigra MRI markers are lower in Alzheimer's disease and are linked to general cognitive function
Source: Brain Commun. 2026 Jun 24;8(4):fcag238. doi: 10.1093/braincomms/fcag238 (PMC13390642; doi:10.1093/braincomms/fcag238)
Supplement: fcag238_Supplementary_Data [file fcag238_supplementary_data.pdf]

Supplementary figures

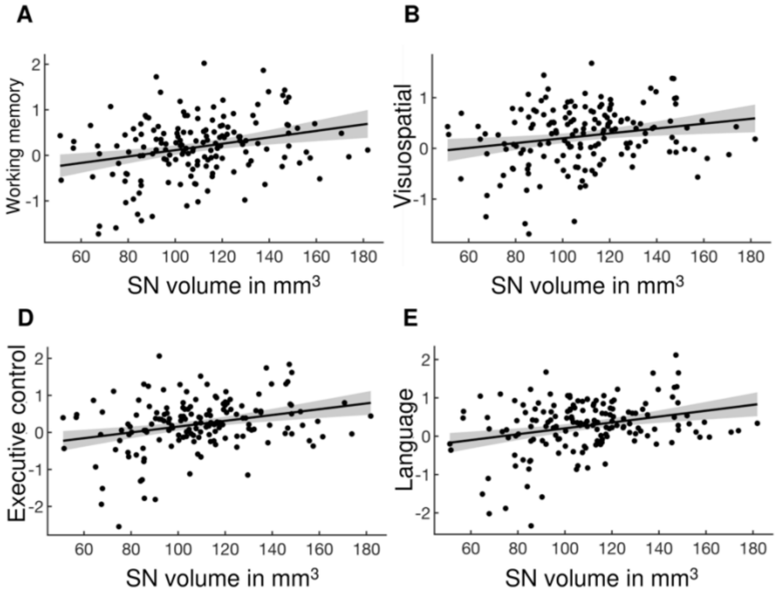

Figure S1: SN volume is associated with the cognitive scores Higher SN volume predicts higher values of all factor scores after correcting for site, years of education, sex, and age. (A) SN volume is positively associated with working memory (R^2 = 0.09, p=0.001, q=0.004, n= 160) (B) SN volume is associated with visuospatial abilities (R^2 = 0.062, p=0.009, q=0.018, n= 160). (C) SN volume is positively associated with memory (R^2 = 0.066, p=0.006, q=0.014, n= 160) (D). Higher SN volume predicts better executive function (R^2 = 0.098, p<0.001, q<0.001, n= 160). (E) higher SN volume predicts a higher language score (R^2 = 0.1, p<0.001, q<0.001, n= 160). All values are corrected for age, sex, years of education, and TIV. The line in each plot represents the best fit, while the shaded area represents one standard deviation.

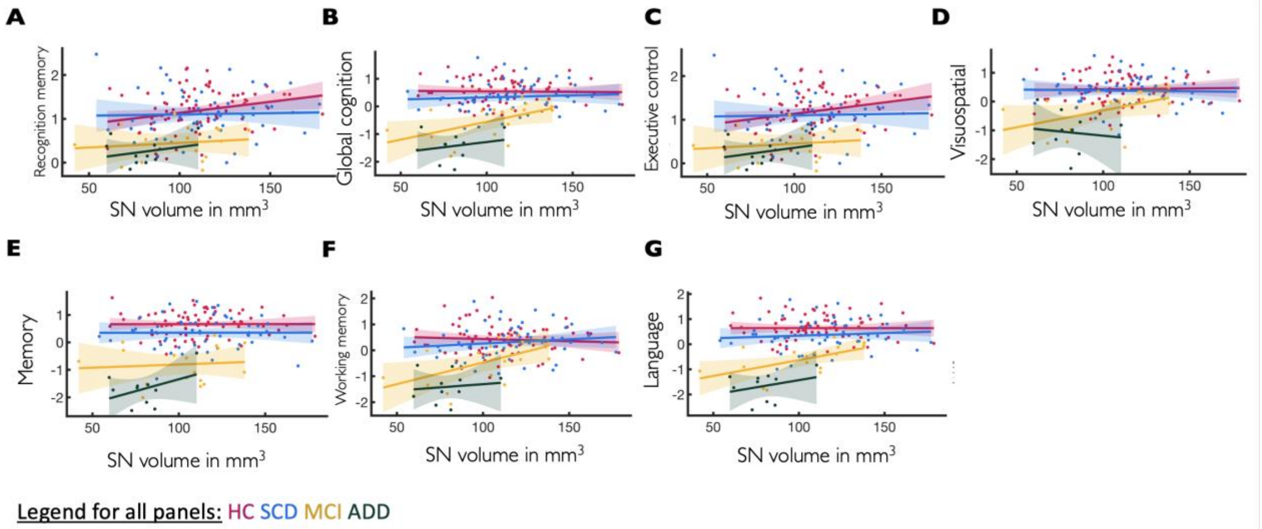

Figure S2: We assessed separate SN volume-cognitive factor associations in different diagnostic group subsets while accounting for age, sex, years of education, and TIV (A) SN volume is associated with recognition memory ($R^2 = 0.071$, $p=0.004$, $q=0.011$, n= 159). Associations between SN volume and recognition memory, examining subgroups of either HC, MCI, SCD, or individuals with Alzheimer‘s disease dementia, were not significant ($p < 0.1$). (B) SN volume is associated with global cognition ($R^2 = 0.096$, $p<0.001$, $q<0.001$, n=160). Associations between SN volume and global cognition, examining subgroups of either HC, MCI, SCD, or individuals with Alzheimer‘s disease dementia, were not significant ($p < 0.1$). (C) SN volume is associated with the executive control factor ($R^2 = 0.098$, $p<0.001$, $q<0.001$, n= 160). Associations between SN volume and the executive control factor, taking subgroups of either HC, MCI, SCD, or individuals with Alzheimer‘s disease dementia, were not significant ($p<0.1$) (D) SN volume is associated with the visuospatial abilities factor ($R^2 = 0.062$, $p=0.009$, $q=0.018$, n= 160). Associations between SN volume and the visuospatial factor taking subgroups of either HC, MCI, SCD or individuals with Alzheimer‘s disease dementia were not significant ($p<0.1$). (E) SN volume is associated with with the memory factor ($R^2 = 0.066$, $p=0.006$, $q=0.014$, n= 160), Associations between SN volume and the memory factor taking subgroups of either HC, MCI, SCD or individuals with Alzheimer‘s disease dementia were not significant ($p<0.1$) (F) SN volume is associated with with the working memory factor ($R^2 = 0.09$, $p=0.001$, $q=0.004$, n= 160), Associations between SN volume and the working memory factor taking subgroups of either HC, MCI, SCD or individuals with Alzheimer‘s disease dementia were not significant ($p<0.1$). (G) SN volume is associated with with the language factor ($R^2 = 0.1$, $p<0.001$, $q<0.001$, n= 160), Associations between SN volume and the language factor taking subgroups of either HC, MCI, SCD or individuals with Alzheimer‘s disease dementia were not significant ($p<0.1$).

Legend: HC=healthy control subjects, SCD=subjective cognitive decline, MCI=mild cognitive impairment, ADD=individuals with Alzheimer‘s disease dementia . The plots are color coded by diagnostic group, Lines denote the fit for each subgroup and shaded area denotes 1STD

	**SN MRI marker associations**	
**Regressor**	**SN contrast**	**SN volume**
**SN MRI measures**		
SN volume	$R^2 = 0.03$ $p=0.2203$ n= 160	
Left vs right side	$t(318)=-2.62, p=0.0092$	$t(318)=5.6$, $p<0.0001$
**Covariates**		

Age	R^2 = 0.078, p=0.006, q=0.084, n= 160	R^2 = 0.042, p=0.199, q=0.279, n= 160
Gender	t(158)=0.47, p=0.638, q= 0.893	t(158)=-1.3, p=0.194, q= 0.279
Years of education	R^2 = 0.005, p=0.811, q=0.964, n= 160	R^2 = 0.032, p=0.036, q=0.063, n= 160
Total intracranial volume	R^2 = 0.356, p=0.125, q=0.583, n= 160	R^2 = 0.351, p=0.254, q=0.323, n= 160
**Cognitive measures**		
Dprime(recognition memory)		
All subjects	R^2 = 0.027, p=0.964, q=0.964, n= 159	R^2 = 0.071, p=0.004, q=0.011, n= 159
All subjects correcting for diagnostic group		R^2 = 0.088, p=0.0071, n= 159
All subjects correcting for hippocampal volume		R^2 = 0.065, p=0.004, n= 159
All subjects correcting for gray matter volume		R^2 = 0.107, p=0.016, n= 159
Only HC		R^2 = 0.082, p=0.018, n= 79
Only SCD		R^2 = 0, p=0.819, n= 54
Only MCI		R^2 = 0.033, p=0.706, n= 16
Only ADD		R^2 = 0.476, p=0.162, n= 10
NPT global cognitive score		
All subjects	R^2 = 0.03, p=0.453, q=0.802, n= 160	R^2 = 0.096, p<0.001 q<0.001, n= 160

All subjects correcting for diagnostic group		R^2 = 0.093, p=0.018, n= 160
All subjects correcting for hippocampal volume		R^2 = 0.091, p<0.001 n= 160
All subjects correcting for gray matter volume		R^2 = 0.112, p=0.007, n= 160
Only HC		R^2 = 0.009, p=0.951, n= 79
Only SCD		R^2 = 0.006, p=0.562, n= 54
Only MCI		R^2 = 0.157, p=0.513, n= 17
Only ADD		R^2 = 0.237, p=0.433, n= 10
<u>Cognitive factor scores</u>		
Visual-spatial abilities		
All subjects	R^2 = 0.049, p=0.062, q=0.434, n= 160	R^2 = 0.062, p=0.009, q=0.018, n= 160
All subjects correcting for diagnostic group		R^2 = 0.076, p=0.104, n= 160
All subjects correcting for hippocampal volume		R^2 = 0.056, p=0.008, n= 160
All subjects correcting for gray matter volume		R^2 = 0.089, p=0.059, n= 160
Only HC		R^2 = 0.011, p=0.659, n= 79
Only SCD		R^2 = -0.001, p=0.912, n= 54
Only MCI		R^2 = 0.125, p=0.849, n= 17

Only ADD		R^2 = 0.134, p=0.681, n= 10
Memory factor		
All subjects	R^2 = 0.028, p=0.588, q=0.893, n= 160	R^2 = 0.066, p=0.006, q=0.014, n= 160
All subjects correcting for diagnostic group		R^2 = 0.068, p=0.25, n= 160
All subjects correcting for hippocampal volume		R^2 = 0.06, p=0.006, n= 160
All subjects correcting for gray matter volume		R^2 = 0.091, p=0.048, n= 160
Only HC		R^2 = 0.009, p=0.826, n= 79
Only SCD		R^2 = 0.003, p=0.656, n= 54
Only MCI		R^2 = 0.122, p=1, n= 17
Only ADD		R^2 = 0.527, p=0.128, n= 10
Working memory factor		
All subjects	R^2 = 0.035, p=0.262, q=0.802, n= 160	R^2 = 0.09, p=0.001, q=0.004, n= 160
All subjects correcting for diagnostic group		R^2 = 0.095, p=0.015, n= 160
All subjects correcting for hippocampal volume		R^2 = 0.085, p=0.001, n= 160
All subjects correcting for gray matter volume		R^2 = 0.105, p=0.012, n= 160

Only HC		R^2 = 0.01, p=0.745, n= 79
Only SCD		R^2 = 0.032, p=0.202, n= 54
Only MCI		R^2 = 0.127, p=0.793, n= 17
Only ADD		R^2 = 0.108, p=0.804, n= 10
Executive functions		
All subjects	R^2 = 0.03, p=0.454, q=0.802, n= 160	R^2 = 0.098, p<0.001, q<0.001, n= 160
All subjects correcting for diagnostic group		R^2 = 0.093, p<0.001 n= 160
All subjects correcting for hippocampal volume		R^2 = 0.114, p=0.005, n= 160
All subjects correcting for gray matter volume		R^2 = 0.011, p=0.659, n= 79
Only HC		R^2 = 0.013, p=0.411, n= 54
Only SCD		R^2 = 0.266, p=0.169, n= 17
Only MCI		R^2 = 0.459, p=0.175, n= 10
Only ADD		R^2 = 0.097, p=0.013, n= 160
Language factor		
All subjects	R^2 = 0.027, p=0.869, q=0.964, n= 160	R^2 = 0.1, p<0.001, q<0.001, n= 160
All subjects correcting for diagnostic group		R^2 = 0.095, p=0.016, n= 160

All subjects correcting for hippocampal volume		R^2 = 0.095, p<0.001 n= 160
All subjects correcting for gray matter volume		R^2 = 0.117, p=0.004, n= 160
Only HC		R^2 = 0.009, p=0.994, n= 79
Only SCD		R^2 = 0.007, p=0.516, n= 54
Only MCI		R^2 = 0.224, p=0.253, n= 17
Only ADD		R^2 = 0.34, p=0.288, n= 10
**Alzheimer's disease markers**		
ANCOV<A F-test between diagnostic groups	F(156,4)= 3.5166, p= 0.223, n=160	F(156,4)= 5.6665, p=0.0010, n=160
Posthoc test: HC vs SCD		P=0.8185
Posthoc test: HC vs MCI		P=0.6
Posthoc test: HC vs Alzheimer's disease dementia		P=0.016
Posthoc test: SCD vs MCI		P=0.3
Posthoc test: SCD vs Alzheimer's disease dementia		P=0.004
Amyloid 42/40 ratio	R^2 = 0.091, p=0.895, q=0.964, n= 71	R^2 = -0.047, p=0.768, q=0.768, n= 71
totaltau	R^2 = 0.101, p=0.375, q=0.802, n= 71	R^2 = -0.03, p=0.288, q=0.336, n= 71
phosphotau181	R^2 = 0.098, p=0.458, q=0.802, n= 71	R^2 = -0.035, p=0.355, q=0.382, n= 71

Supplementary table S1: Overview of all associations tested in this work. Unless otherwise specified, all statistics are the main effect of the association: variable of interest ~ SN MRI factor + age + sex + years of education + TIV, p: uncorrected p value, q: FDR-corrected p value.

	**Diagnostic group differences**
**Dependent variable**	**Statistics**
**Covariates**	
Age	F(3,156)=6.04, p=0
Sex	F(3,156)=3.07, p=0.03
Years of education	F(3,156)=2.52, p=0.06
TIV	F(3,156)=1.89, p=0.13
**Cognitive parameters**	
Recognition memory	F(3,156)=19.52, p=0
Global cognition	F(3,156)=59.72, p=0
Working memory	F(3,156)=31.14, p=0
Language	F(3,156)=69.56, p=0
Memory	F(3,156)=85.06, p=0
Executive funciton	F(3,156)=44.93, p=0
Visual	F(3,156)=28.24, p=0
**CSF measures**	
Total tau	F(3,156)=14.81, p=0
Amyloid 42/40	F(3,156)=11.87, p=0
Phosphotau	F(3,156)=10.81, p=0

Supplementary table S2. One-way ANCOVA results comparing the dependent variables between diagnostic groups while account for age, sex, years of education and TIV,
